# Acid resistance of Masson pine (*Pinus massoniana* Lamb.) families and their root morphology and physiological response to simulated acid deposition

**DOI:** 10.1038/s41598-020-79043-1

**Published:** 2020-12-16

**Authors:** Sijie Zhou, Min Zhang, Shuzhan Chen, Wen Xu, Liting Zhu, Shurui Gong, Xiaoqin He, Ping Wang

**Affiliations:** 1 College of Biology and the Environment, Nanjing Forestry University, Nanjing, 210037 China; 2 Key Laboratory of Forest Genetics and Biotechnology of Ministry of Education, Nanjing Forestry University, Nanjing, 210037 China

**Keywords:** Ecology, Environmental sciences

## Abstract

*Pinus massoniana* Lamb. is one of the most sensitive species to acid deposition among forest woody plants, but differences in acid resistance among pine families still exist. It is of great significance to study the differences in acid resistance of Masson pine families and to analyze the physiological regulation mechanism of their acid resistance. In this study, the 100-day-old seedlings of 16 Masson pine families were treated with the simulated acid rain (SAR) at different pH levels (5.6, 4.5, 3.5 and 2.5) for 100 days to investigate the plant morphology, chlorophyll content, and root physiological responses. Results showed that pine family No. 35 maintained the good morphology, high chlorophyll content and organic acids secretion, and low plasma membrane permeability exposed to SAR, while family No. 79 presented the opposite. SAR not only increased the root plasma membrane permeability, but also induced an exudation of organic acids from the pine roots, and the test parameters changed sharply when the SAR pH was lower than 4.5. The results indicated that Masson pine could resist to acidic environment (pH 4.5–5.6), and family No. 35 had the acid resistance while the family No. 79 was sensitive to acid stress. The acid resistance diversity of different pine families had close relation with the root physiological processes, including the root plasma membrane permeability and organic acids secretion. For the future research, the natural genetic variation of Masson pine in response to acid stress and its acid resistance mechanism should be further studied.

## Introduction

The rapid growth of economy and population has resulted in the excessive consumption of fossil energy, and it consequently increased the emission of SO_x_ and NO_x_, leading to the atmospheric acid deposition. Acid rain pollution has become one of the global top ten serious environmental challenges since the 1970s^1^. After Europe and North America, China is the third-largest heavy acid deposition region in the world^2^. Approximately 40% of China suffered from acid precipitation, and the incidence of acid rain in cities reached up to 19.8% in 2016^3,4^. Busch et al.^5^ (2001) predicted that acid deposition in forest ecosystems would be about 46% higher in 2040 than that was in the 1980s.

Acid deposition not only seriously threatens the biochemical cycle of the earth, but also destroys the stability of terrestrial ecosystems^6^. As the important part of the terrestrial ecosystem, plants can be considered as the main victim of acid deposition pollution^7^. Previous studies found that the damage mechanism of acid deposition on plants mainly involved destroying the ultrastructure of chloroplasts, inhibiting photosynthetic capacity, inducing the membrane lipid peroxidation, restraining respiration and other physiological metabolisms^8–10^. The soil acidification and the release of soil base ions caused by acid rain erosion can seriously damage plant roots, such as inhibiting root elongation and reducing the number of lateral roots^7^, thereby weakening their ability to absorb and transport water and nutrients^11^. These inhibiting effects will eventually cause the wilting, fading and death of plants.

Masson pine (*Pinus massoniana* Lamb.) is a native tree species in China with a wide distribution range and large forest area. It is characterized by drought resistance, rapid growth, strong adaptability and high economic value and is a pioneer tree species for afforestation and reforestation^12^. However, large-scale observations showed that the regions that are seriously polluted by acid deposition are concentrated mainly in the south and the southwest of China, especially in city-clusters such as the Yangtze River Delta area, the Pearl River Delta area, and the Sichuan Basin^13^. Where the annual average pH of precipitation has been generally below 4.5 in recent years, even as low as 3.0^14^. Wu et al.^15^ (1998) reported that Masson pine was sensitive to acid deposition, and the yield decreased by 43% when exposed to acid rain of pH ≤ 4.0. But Quan and Ding^16^ (2017) claimed that Masson pine showed the strong adaptability to the acidic soils in south China. Under the long-term natural selection and reproductive isolation, Masson pine had abundant intra species natural genetic variation. He et al.^17^ (2013) reported that the physiological responses of Masson pine to different levels of phosphorus stress exhibited highly significant variation among 11 elite pine families. But the differences in acid resistance of Masson pine families is not well-understood. Therefore, studying the differences in acid resistance of the pine families and finding scientific ways to alleviate the damage on Masson pine caused by acid deposition is of great significance for the restoration of forest ecosystems eroded by acid deposition.

When plants are subjected to stress, the first response is the plasma membrane and the functional protein cells expressed on the plasma membrane^18^. Consequently, the relative permeability of root plasma membrane can directly reflect the stability of plant root cells internal environment and the adaptability and resistance of plant to external soil environment changes. Moreover, acid rain has also been reported to affect the plant chlorophyll content and photosynthesis as well^19^.

Low molecular weight organic acids (e.g., oxalic acid (OA), malic acid (MA), citric acid (CA), etc.) are important components of root exudate, which play a key role in plant vivo osmosis, pH regulation, ion balance, and other rhizospheric reactions in response to environmental stress^20^. Root organic acids can also act in reducing the high oxidation state of some metal elements [e.g., Fe (III) and Cr (VI), etc.], so that they can be better absorbed and utilized by plants^21^. Besides, Zhang et al.^22^ (2018) found that citric acid and fumaric acid significantly stimulated *Hansschlegelia zhihuaiae* S113 colonization on cucumber roots, which allowed for improved cucumber growth. However, both the types and contents of organic acids secretion are affected by plant environmental stress and plant species^23^. Since the genetic variation of Masson pine is complex and diverse, the responses of different families to environmental stress are also different. Hence, studying the organic acids secretion of Masson pine families with different acid resistance under acid deposition is very important to further understand the physiological mechanism of plant acid resistance.

In this work, 100-day-old seedlings of 16 Masson pine families were used as the test materials to study the response of plant morphology, photosynthetic traits, and root physiological parameters of Masson pine families to the simulated acid rain with different pH values. The purpose of this study is to investigate the differences in the acid resistance of pine families and to identify the key physiological factors of Masson pine in response to simulated acid deposition.

## Results

### Changes in morphology indexes of Masson pine families

**Figure 1:**
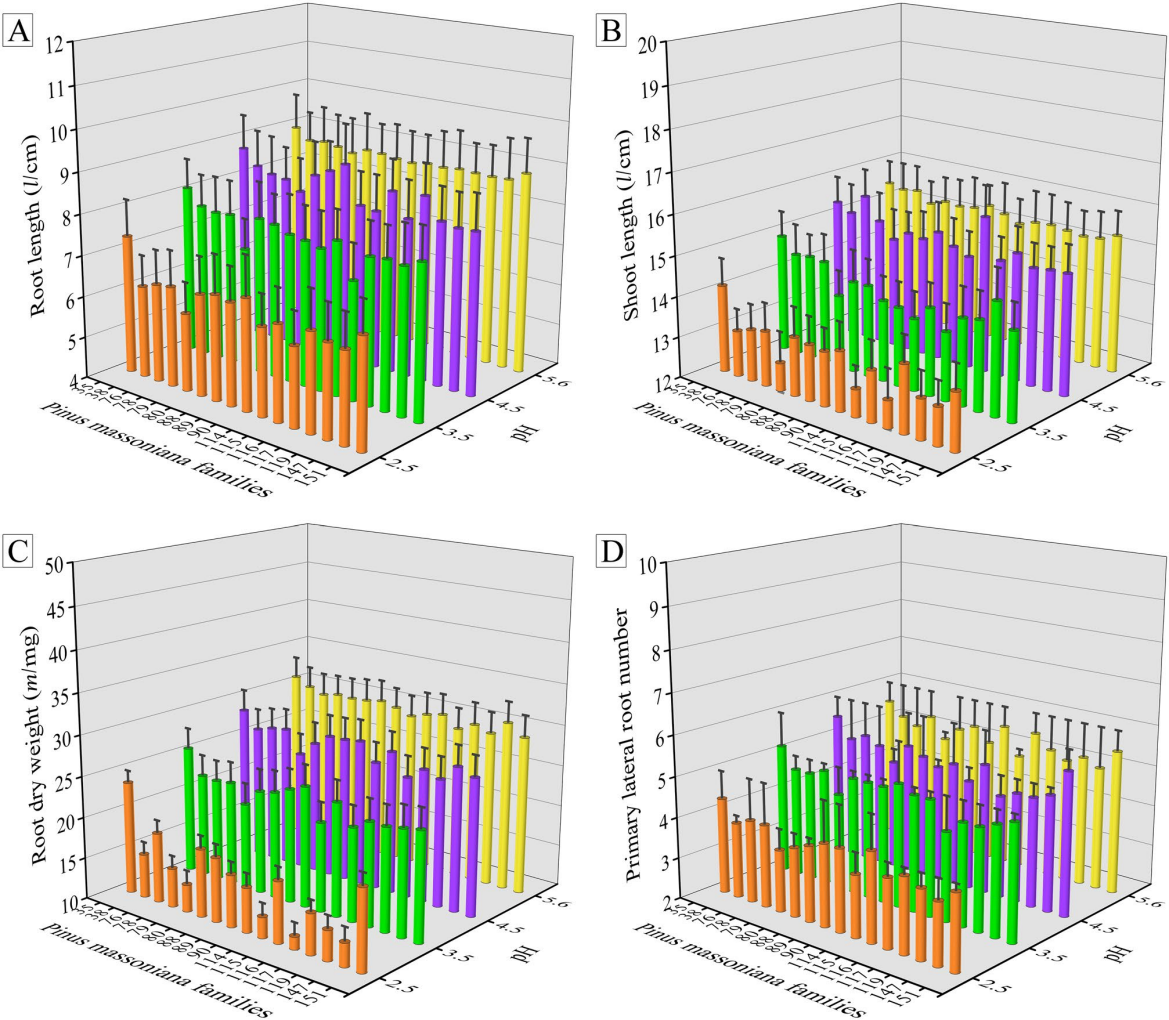
The morphology indexes, (**A**) root length (*l*/cm), (**B**) shoot length (*l*/cm), (**C**) root dry weight (*m*/mg), and (**D**) primary lateral root number of Masson pine families exposed to the SAR at pH of 2.5, 3.5, 4.5 and 5.6. Results are expressed as mean ± standard error (n = 3).

When exposed to simulated acid rain of different pH, the family No. 35 has always had longer root and shoot length, heavier root dry weight and more primary lateral root number among all 16 pine families, while families Nos. 79, 114 and 116 showed the opposite performance (Fig. 1). Although the length of roots and shoots (Fig. 1A,B), the dry weight of roots (Fig. 1C), and the number of primary lateral roots (Fig. 1D) showed different degrees of decrease with the decrease of pH value of SAR, the pine morphology was slightly effected by the SAR at pH 5.6–4.5, while under the acid rain gradient of pH 2.5–3.5, the pine morphology indexes decreased obviously.

### Changes of chlorophyll contents for the pine seedlings

**Figure 2:**
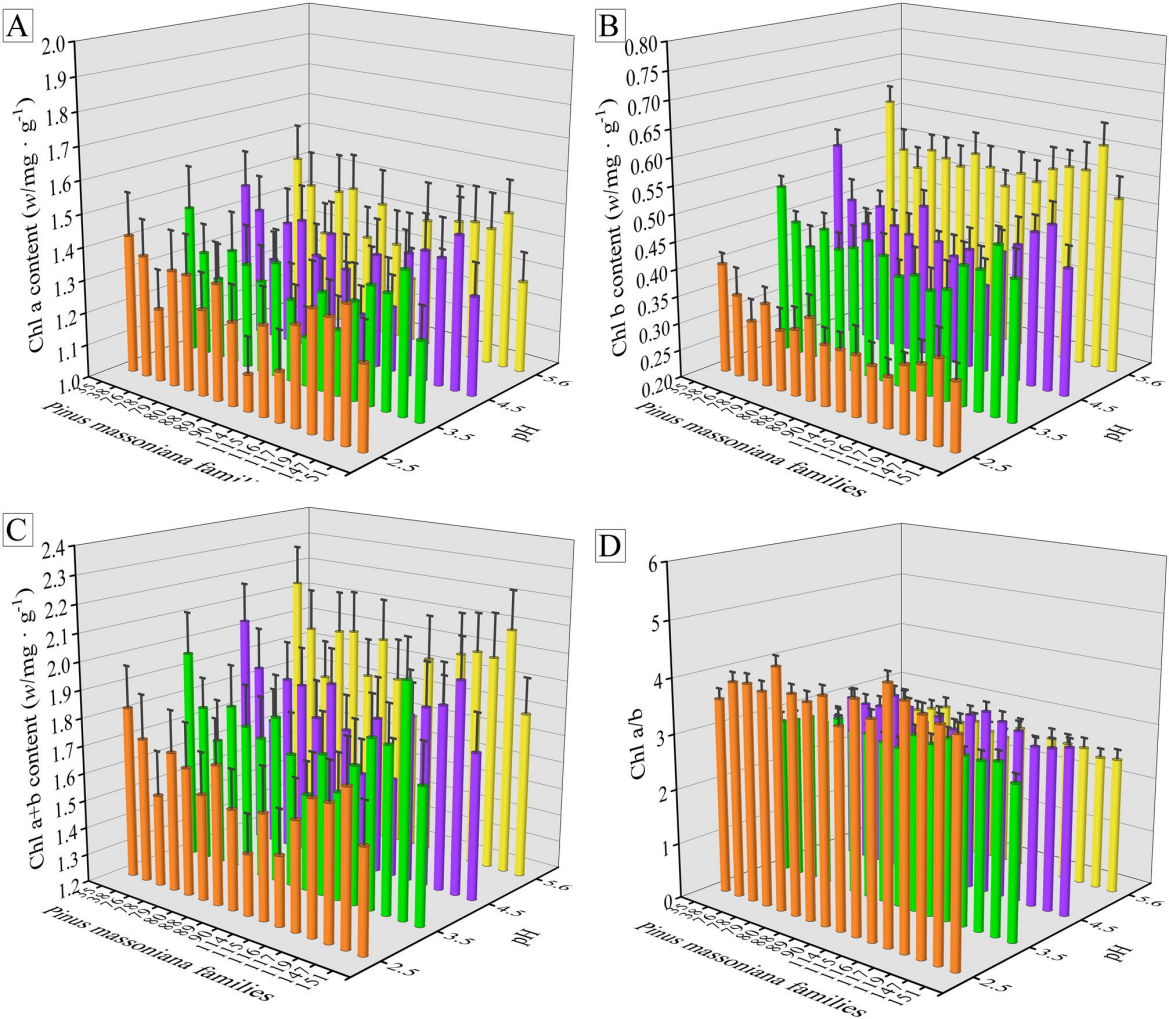
Contents of (**A**) chlorophyll *a* (*w*/mg g^−1^), (**B**) chlorophyll *b* (*w*/mg g^−1^), (**C**) chlorophyll *a* + *b* (*w*/mg g^−1^), and (**D**) the value of chlorophyll *a*/*b* for the Masson pine families exposed to the SAR at pH of 2.5, 3.5, 4.5, and 5.6, respectively. Results are expressed as mean ± standard error (n = 3).

The contents of chlorophyll *a*, chlorophyll *b* and chlorophyll *a* + *b* decreased with the increase of acid intensity, however, the value of chlorophyll *a*/*b* increased (Fig. 2). In addition, the chlorophyll *b*, *a* + *b* contents of the all families under severe acid stress of pH 2.5 were lower than those under moderate (pH 3.5), mild (pH 4.5) acid treatments and control treatment (pH 5.6), and the chlorophyll *a*/*b* value under severe acid stress was significantly higher than those of the other three treatments. Moreover, treated with the SAR of different pH, family No. 35 had higher contents of chlorophyll *a*, chlorophyll *b*, chlorophyll *a* + *b* and lower value of chlorophyll *a*/*b*, while the opposite phenomena were observed in the families Nos. 79, 114, and 116.

### Changes in the relative permeability of root plasma membrane

**Figure 3:**
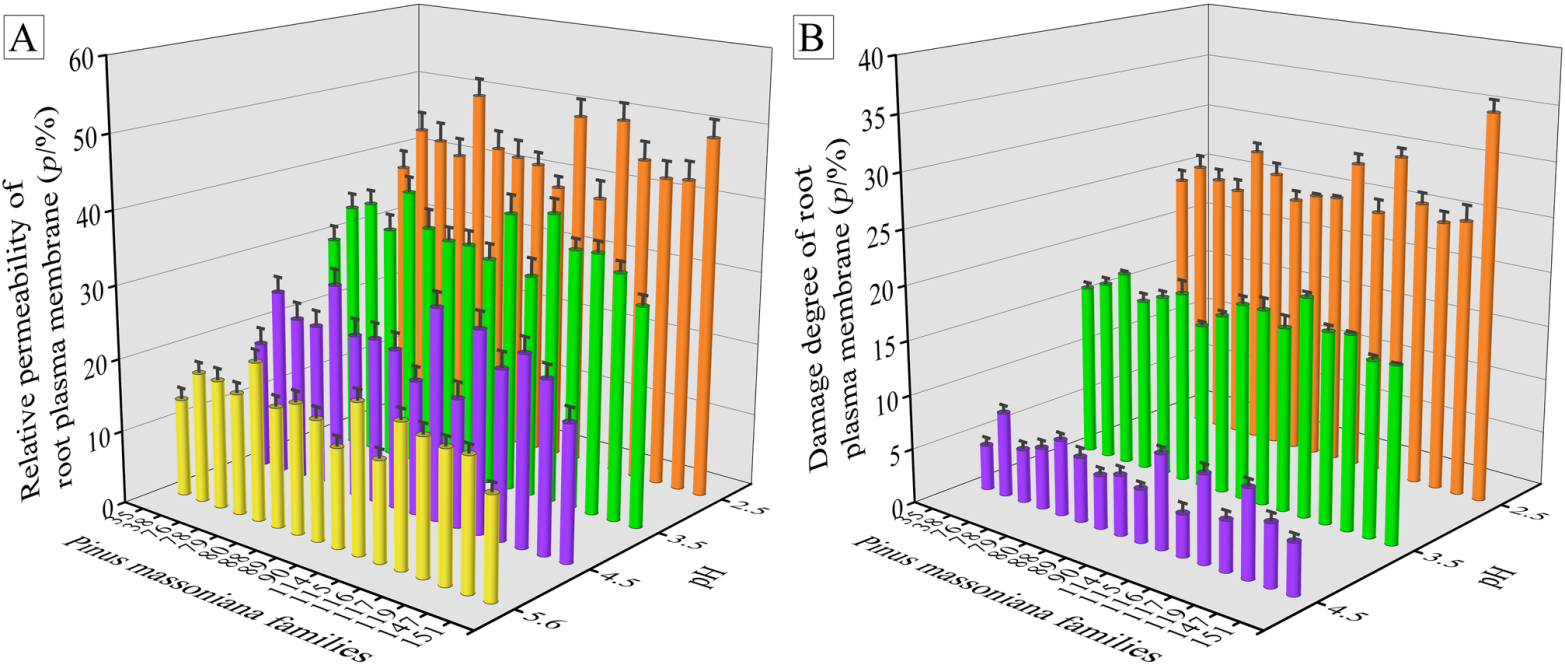
(**A**) The relative permeability (*p*/%) and (**B**) damage degree (*p*/%) of root plasma membrane of 16 Masson pine families exposed to the SAR. The value of membrane damage degree of Masson pine families at pH 5.6 is 0, which is not shown in (**B**). Results are expressed as mean ± standard error (n = 3).

The permeability and the damage degree of root plasma membrane of the Masson pine families both increased with the increase of acid intensity (Fig. 3). In addition, Masson pine family No. 35 kept low permeability and damage degree of root plasma membrane among all test pine families, while families Nos. 79, 114 and 116 presented opposite performances.

### Changes in the root secretion of organic acids and the rhizosphere pH of Masson pine family No. 35 and No. 79

**Figure 4:**
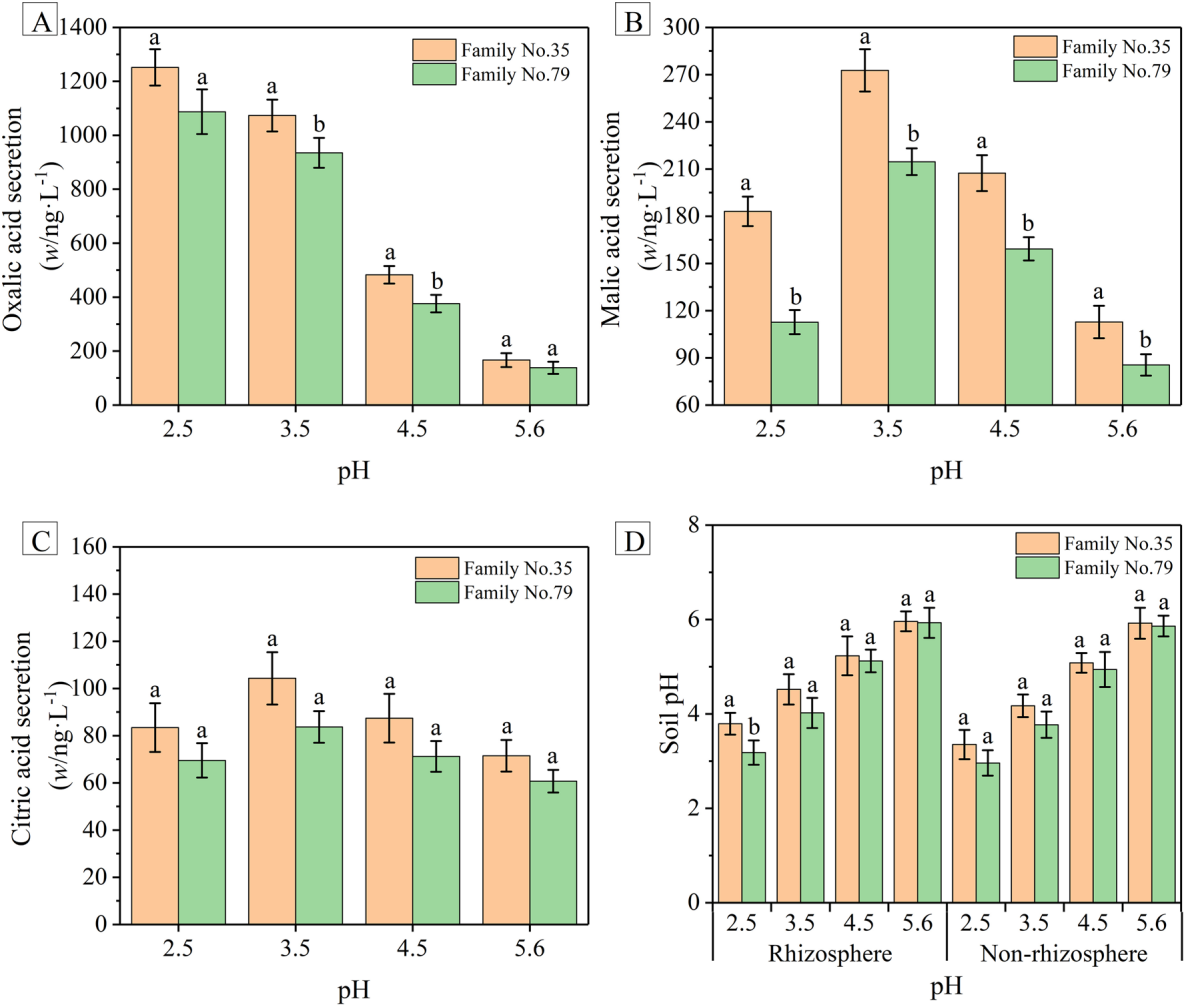
Root organic acids secretion, (**A**) oxalic acid (*w*/ng L^−1^), (**B**) malic acid (*w*/ng L^−1^), (**C**) citric acid (*w*/ng L^−1^), and (**D**) soil pH of *Pinus massoniana* families Nos. 35 and 79. Based on Duncan’s multiple range tests with *P* < 0.05, significant differences between Masson pine family No. 35 and No. 79 are represented by lowercase letters. Results are expressed as mean ± standard error (n = 3).

In order to reflect the difference of root organic acids secretion of Masson pine families with different acid resistance strongly and intuitively, the pine family No. 35 and No. 79 were selected as the acid-resistant and acid-sensitive species respectively, to further study their root physiological response to the SAR (Fig. 4). The results of the ANOVA are tabulated in Table 1.

**Table 1:** ANOVA results of root organic acids secretion and soil pH of Masson pine exposed to the SAR (at 95% confidence level) (n = 3).

SAR pH	Source	SS	DF	MS	F	*P*
**Oxalic acid secretion**						
2.5	Masson pine family	40,639.740	1	40,639.740	7.153	0.056
	Error	22,725.000	4	5681.250		
	Total	63,364.740	5			
3.5	Masson pine family	28,731.840	1	28,731.840	8.820	0.041
	Error	13,029.700	4	3257.425		
	Total	41,761.540	5			
4.5	Masson pine family	17,109.360	1	17,109.360	16.050	0.016
	Error	4264.100	4	1066.025		
	Total	21,373.460	5			
5.6	Masson pine family	1209.840	1	1209.840	2.100	0.221
	Error	2304.020	4	576.005		
	Total	3513.860	5			
**Malic acid secretion**						
2.5	Masson pine family	7434.240	1	7434.240	100.701	0.001
	Error	295.300	4	73.825		
	Total	7729.540	5			
3.5	Masson pine family	5063.415	1	5063.415	40.216	0.003
	Error	503.620	4	125.905		
	Total	5567.035	5			
4.5	Masson pine family	3484.860	1	3484.860	37.731	0.004
	Error	369.440	4	92.360		
	Total	3854.300	5			
5.6	Masson pine family	1117.935	1	1117.935	14.678	0.019
	Error	304.660	4	76.165		
	Total	1422.595	5	7434.240		
**Citric acid secretion**						
2.5	Masson pine family	289.815	1	289.815	3.637	0.129
	Error	318.760	4	79.690		
	Total	608.575	5			
3.5	Masson pine family	636.540	1	636.540	7.573	0.051
	Error	336.200	4	84.050		
	Total	972.740	5			
4.5	Masson pine family	393.660	1	393.660	5.308	0.083
	Error	296.680	4	74.170		
	Total	690.340	5			
5.6	Masson pine family	174.960	1	174.960	5.151	0.086
	Error	135.860	4	33.965		
	Total	310.820	5	289.815		
**Rhizospheric soil pH**						
2.5	Masson pine family	0.558	1	0.558	9.264	0.038
	Error	0.241	4	0.060		
	Total	0.799	5			
3.5	Masson pine family	0.375	1	0.375	3.662	0.128
	Error	0.410	4	0.102		
	Total	0.785	5			
4.5	Masson pine family	0.018	1	0.018	0.161	0.709
	Error	0.451	4	0.113		
	Total	0.470	5			
5.6	Masson pine family	0.001	1	0.001	0.018	0.899
	Error	0.293	4	0.073		
	Total	0.294	5			
**Non-rhizospheric soil pH**						
2.5	Masson pine family	0.228	1	0.228	2.700	0.176
	Error	0.338	4	0.085		
	Total	0.566	5			
3.5	Masson pine family	0.240	1	0.240	3.529	0.133
	Error	0.272	4	0.068		
	Total	0.512	5			
4.5	Masson pine family	0.029	1	0.029	0.325	0.599
	Error	0.362	4	0.090		
	Total	0.391	5			
5.6	Masson pine family	0.005	1	0.005	0.069	0.806
	Error	0.315	4	0.079		
	Total	0.320	5			

SS is the sum of squares, DF is the degrees of freedom, and MS is the mean square.

The secretion of root organic acids of Masson pine increased with the pH value of SAR decreased from 5.6 to 3.5, and the secretion of oxalic acid and malic acid was obviously higher than that of citric acid according to Fig. 4A–C. No succinic acid and acetic acid were detected (Table S1). The root organic acids (oxalic acid, malic acid, and citric acid) secretion of Masson pine family No. 35 was higher than that of No. 79 exposed to the SAR of different acidity. Specifically, the root oxalic acid secretion of pine family No. 35 was significantly higher than that of No. 79 treated with the SAR at pH 3.5 (*P* = 0.041 < 0.05, Table 1) and 4.5 (*P* = 0.016 < 0.05, Table 1). Similarly, the root malic acid secretion of pine family No. 35 was significantly higher than that of No. 79 treated with the SAR at pH 2.5 (*P* = 0.001 < 0.05, Table 1), 3.5 (*P* = 0.003 < 0.05, Table 1) and 4.5 (*P* = 0.004 < 0.05, Table 1).

The comparison between the soil background pH (6.65, Table S3) and the soil pH of Masson pine seedlings subjected to SAR (Fig. 4D, Table S2) showed that the rhizosphere and non-rhizosphere pH were lower and decreased with the decrease of SAR pH. Figure 4D also showed that although the soil pH of family No. 35 decreased with the decrease of SAR pH, it was still higher than that of No. 79 treated with SAR at the same acidity level. Particularly, the rhizospheric soil pH of family No. 35 treated with SAR of pH 2.5 was significantly higher than that of No. 79 (*P* = 0.038 < 0.05, Table 1).

### Correlation coefficients, principle component analysis (PCA), and cluster analysis

Correlations among the morphology indexes, photosynthetic traits, and root physiological parameters of Masson pine families subjected to SAR were revealed by Pearson’s correlation coefficients. The significant negative correlations between oxalic acid secretion and root length (− 0.726*), shoot length (− 0.709*), rhizosphere pH (− 0.924**), and non-rhizosphere pH (− 0.948**) were observed (Table 2). The root plasma membrane permeability was significantly negatively correlated with photosynthetic traits (except chlorophyll *a*/*b*), morphology indexes, rhizospheric and non-rhizospheric soil pH (*P* < 0.01, Table 2). The rhizospheric soil pH was significantly negatively correlated with the chlorophyll *a*/*b* (− 0.860**, Table 2).

**Table 2:** Correlation coefficients (*r*) among the photosynthetic traits, morphology indexes, and root physiological parameters for the pine seedlings of families Nos. 35 and 79, which were exposed to the SAR at pH of 5.6, 4.5, 3.5, and 2.5.

Variables	Chl. *a*/*b*	RPRPM	OA	MA	CA
Chl. *a*	− 0.726*	− 0.865**	− 0.551	− 0.099	0.052
Chl. *b*	− 0.959**	− 0.966**	− 0.693	0.021	0.087
Chl. *a* + *b*	− 0.899**	− 0.959**	− 0.661	− 0.026	0.076
Chl. *a*/*b*	1	0.928**	0.650	− 0.172	− 0.170
RPRPM	0.928**	1	0.798*	0.077	0.046
RL	− 0.935**	− 0.982**	− 0.726*	0.027	0.049
SL	− 0.936**	− 0.966**	− 0.709*	0.063	0.071
RDW	− 0.931**	− 0.958**	− 0.698	− 0.012	0.009
PLRN	− 0.946**	− 0.943**	− 0.584	0.242	0.286
OA	0.650	0.798*	1	0.529	0.570
MA	− 0.172	0.077	0.529	1	0.959**
CA	− 0.170	0.046	0.570	0.959**	1
RpH	− 0.860**	− 0.943**	− 0.924**	− 0.280	− 0.291
NRpH	− 0.836**	− 0.929**	− 0.948**	− 0.331	− 0.342

**, and * = significant at 0.01 and 0.05 levels, respectively. The underlined data indicates a significant negative correlation between the two indicators.

*Chl. a* chlorophyll *a*, *Chl. b* chlorophyll *b*, *Chl. a* + *b* chlorophyll *a* + *b*, *Chl. a*/*b* chlorophyll *a*/*b*, *RPRMP* relative permeability of plasma membrane, *RL* root length, *SL* shoot length, *RDW* root dry weight, *PLRN* primary lateral root number, *OA* oxalic acid, *MA* malic acid, *CA* citric acid, *RpH* rhizosphere pH, *NRpH* non-rhizosphere pH. The abbreviations above are the same as the following.

**Figure 5:**
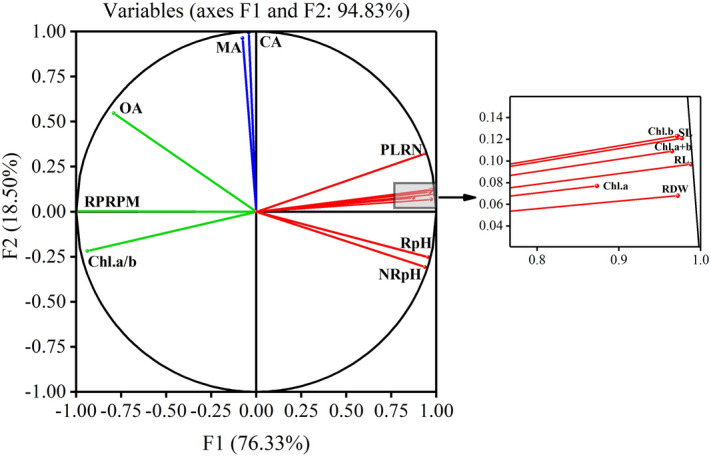
Principle component analysis (PCA) showing the associations among the photosynthetic traits, morphology indexes, and root physiological parameters of Masson pine families Nos. 35 and 79.

In PCA, the principal components 1 and 2 accounted for 94.83% of the observed variables (F1 = 76.33% and F2 = 18.50%, Fig. 5). F1 was characterized by high positive scores for photosynthetic traits (except chlorophyll *a*/*b*), morphology indexes, rhizosphere and non-rhizosphere pH, and high negative scores for chlorophyll *a*/*b*, relative permeability of root plasma membrane, and oxalic acid secretion. F2 was characterized by high positive scores for malic acid and citric acid secretion.

**Figure 6:**
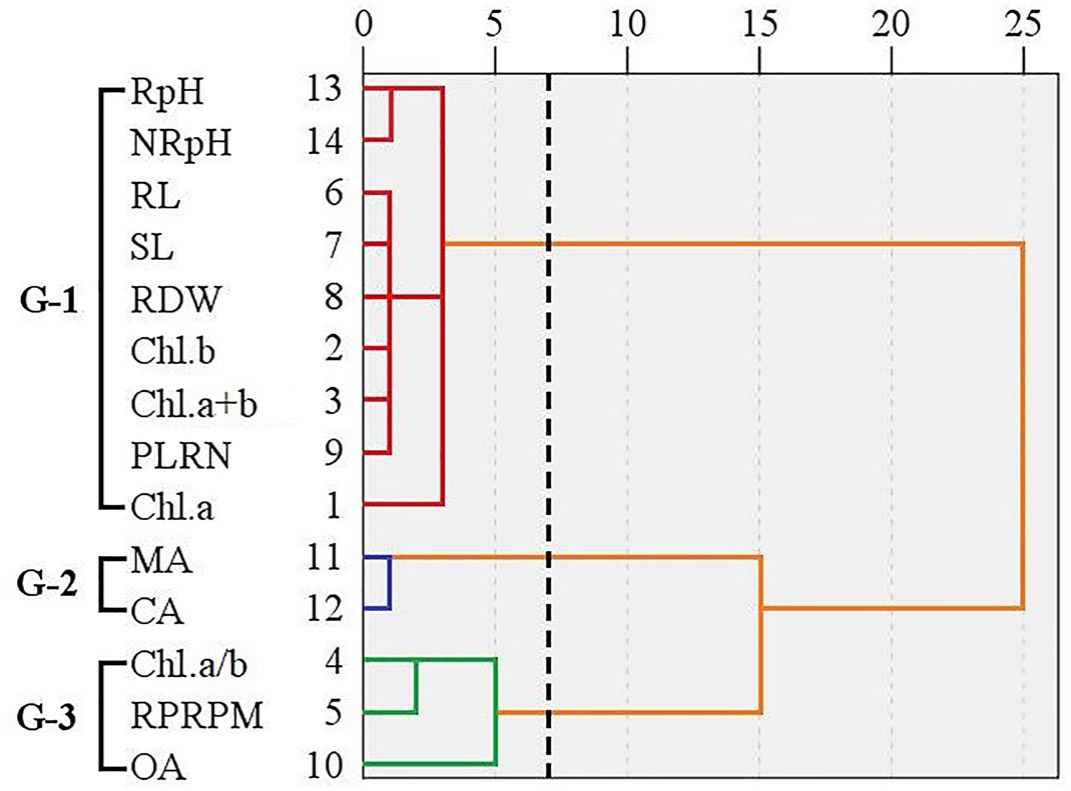
Dendrograms of cluster analysis showing the similarity for the test indicators of Masson pine family No. 35 and No. 79 exposed to the SAR at pH of 5.6, 4.5, 3.5, and 2.5.

Cluster analysis (Fig. 6) demonstrated the groups of plant physiological attributes of Masson pine families, which responded by a similar way to SAR treatment. They were grouped into three distinct sections according to their similarities when exposed to the SAR. The morphology indexes (root length, shoot length, root dry weight, primary lateral root number), photosynthetic traits (chlorophyll *a*, chlorophyll *b*, chlorophyll *a* + *b*), rhizospheric pH and non-rhizospheric pH were affected collectively and were grouped as G-1, which were positively correlated with the SAR pH. And the chlorophyll *a*/*b*, root plasma membrane permeability and oxalic acid were grouped as G-3, they were negatively correlated with the SAR pH. Besides, Malic acid and citric acid secretion exhibited a similar pattern and were grouped as G-2.

## Discussion

### Differences in plant morphology, chlorophyll content, and root plasma membrane permeability of the 16 Masson pine families

Our results in Fig. 1 showed that the pine seedlings morphology was closely related to the SAR acidity. When treated with SAR at pH below 3.5, the pine seedlings showed the phenomenon of shortened root and shoot length, reduced root dry weight and lateral roots. A large amount of H^+^ caused by SAR was a major factor that threatened the growth of trees, which was absorbed into plant cells through roots, inducing intracellular free radical and proton effects^24^, thus adversely affecting the root morphology. Also, acid rain erosion will cause soil acidification, and the leaching of salt-based cations in acidified soil will reduce the ability of soil to supply nutrients^25^, thus affecting the absorption and utilization of nutrients in Masson pine roots, and then leading to the decrease of seedling morphology indexes^26^. Thus, we can infer that Masson pine seedlings had a certain resistance to acid rain of pH 4.5, but had poor resistance to acid rain with pH lower than 3.5.

When plants are exposed to acid rain, the synthesis of chlorophyll can be inhibited, which accordingly leads to negative effects on leaf photosynthesis and metabolism^6,7^. Liu et al.^27^ (2007) studied the changes of chlorophyll in Masson pine under continuous acid rain treatment, indicating that the photochemical efficiency and energy conversion efficiency were decreased in the case of acid rain erosion and strong light. Therefore, plant chlorophyll content can be used as an indicator of plant stress resistance to characterize the physiological situation of plants under environmental stress^28^. High chlorophyll content and low chlorophyll *a*/*b* value are the important traits possessed by excellent Masson pine families^27^. Generally, the higher the chlorophyll content, the stronger the photosynthesis, accordingly producing more photosynthetic products, which can promote the growth of plants. Liang and Zhang^28^ (2018) found that chlorophyll content of soybean (*Glycine max*) seedlings decreased with the application of acid rain (pH 4.5/pH 3.0). Similarly, our results (Fig. 2) showed that treated with SAR, the chlorophyll content in pine needles decreased and the ratio of chlorophyll *a*/*b* increased. Under severe (pH 2.5) and moderate (pH 3.5) SAR treatments, the chlorophyll contents of Masson pine were much lower than that of the control. It indicated that acid rain of pH 2.5 and 3.5 inhibited the synthesis of chlorophyll, causing Masson pine to fail to make full use of the absorbed light energy, weakening photosynthesis, and reducing the resistance of Masson pine to acid rain.

Plant cell membrane plays an important role in regulating the transport of intracellular and extracellular substances and maintaining the normal physiological metabolism of cells, the permeability of which is closely related to the intensity of stress under adversity^29^. Wagatsuma^30^ (2017) proposed that the retardation of aluminum permeation through the plasma membrane lipid bilayer mechanism was a new concept of aluminum tolerance in plants. The results in Fig. 3 showed that the root plasma membrane permeability of Masson pine treated with SAR of pH 5.6 and 4.5 remained low, while the plasma membrane was severely damaged by SAR of pH 3.5 and 2.5. Hence, it can be inferred that SAR can destroy the integrity of plant plasma membrane, but Masson pine seedlings have a certain acid resistance and can maintain the integrity of membrane under mild SAR stress. The similar phenomena were reported by Liang et al.^31^ (2019) who found that the membrane permeability of rice and soybean leaves did not change exposed to SAR at pH 5.0, 4.5 or 4.0, but which increased when treated with SAR at pH 3.5, 3.0 or 2.5.

### Differences in plant morphology and physiological traits of the Masson pine families No. 35 and No. 79

The results in Figs. 1 and 2 showed that the Masson pine family No. 35 maintained better phenotypic morphology, higher content of chlorophyll *a*, chlorophyll *b*, and chlorophyll *a* + *b*, lower chlorophyll *a*/*b* value in response to SAR of different pH among 16 Masson pine families. On the contrary, the pine families Nos. 79, 114 and 116 presented the worse plant morphology and lower chlorophyll content subjected to simulated acid deposition compared with others. In general, plants that are resistant to environmental stress exhibit some good phenotypic and physiological traits to a certain extent^24^. When exposed to adverse environment, plants with good phenotypic morphology and high chlorophyll content can better absorb nutrients from soil and carry out photosynthesis, which is conducive to its growth under environmental stress^26,28^. Therefore, the results indicated that the acid resistance of Masson pine family No. 35 was stronger than that of families Nos. 79, 114 and 116.

When plants are subjected to adversity stress, a large amount of reactive oxygen species are produced to attack the membrane system, leading to the peroxidation of the cytoplasmic membrane and changes in membrane lipid components^32^. Which directly affecting the fluidity and permeability of membrane, resulting in the leakage of intracellular electrolytes and small molecular organic substances^32^. According to Fig. 3, the stability and integrity of membrane of Masson pine families Nos. 79, 114 and 116 was damaged more severely than that of family No. 35 exposed to SAR of the same pH level. Serious damage to the root plasma membrane of Masson pine families Nos. 79, 114 and 116 was likely to cause the leakage of intracellular nutrients and electrolytes, accordingly making the Masson pine root physiological metabolism unable to carry out normally. But the plasma membrane integrity of pine family No. 35 maintained well, which was beneficial to its resistance to acid stress.

Among studied 16 Masson pine families, family No. 35 and families Nos. 79, 114 and 116 showed the significant differences in phenotypic and physiological indexes responding to acid stress (Figs. 1, 2, 3). The metabolic system, especially root organic acids secretion, plays an important role in regulating the response of plants to abiotic stress. In order to reveal the key physiological factors working in mechanism of resistance to acid stress in Masson pine roots, pine families No. 35 and No. 79 were selected as relatively acid-resistant and acid-sensitive plants respectively to observe the differences in root organic acids secretion and soil pH in response to acid stress.

Organic acids are important plant root exudates, which can regulate the osmotic balance of plant cells, intracellular pH and ionic concentration^20^. Root organic acids can interact with some metal ions, such as cadmium (Cd), aluminum (Al)^33^, gallium (Ga), copper (Cu), manganese (Mn) and lead (Pb)^34^, chelated to form organometallic complexes^35^, thereby reducing the toxicity of metal ions to plants^36^. Therefore, root secretion of organic acids can be used as a special protective mechanism for plants to cope with acid stress. We observed that induced by SAR, the root secretion of organic acids increased (Fig. 4A–C), that was the same as Ahmed et al.^37^ (2016) and Wang et al.^38^ (2006). This is probably because the large amount of organic acids secreted by pine roots can react with H^+^ and metal ions activated by SAR to alleviate the injury caused by SAR to plants. In addition, root organic acids can regulate the availability of soil nutrients which are difficult to use, making them easy to be absorbed and utilized by plants^39^. Hence, the increase of root organic acids secretion induced by acid stress is a physiological defense mechanism of plants to against adversity. According to Fig. 4 and Table 1, when treated with SAR of the same acidity, the secretion of organic acids (except citric acid) exuded from pine roots of family No. 35 was obviously higher than that of family No. 79. This is probably because SAR treatment increased the membrane permeability of pine family No. 79, destroyed the integrity of plasma membrane, and adversely affected the root physiological metabolism of Masson pine, resulting in the less secretion of root organic acids of the family No. 79. Thus, the result indicated that the root organic acids secretion also contributed to the acid resistance of Masson pine.

Soil buffer system, plant nutrients and metal cations, respiratory CO_2_ production, relative permeability of roots plasma membrane, root secretion of organic acids and proton can lead to fluctuations in the rhizosphere pH^40^. Due to the existence of buffer systems such as hydroxyl aluminum and exchangeable cations in the soil, which can react with H^+^ to reduce soil acidification and increase the soil pH. According to Table S3 and Fig. 4D, the soil pH of Masson pine seedlings subjected to SAR were lower than the soil background pH and decreased with the decrease of SAR pH. Which illustrated that the simulated acid rain could cause soil acidification and affect the rhizosphere environment of Masson pine. Moreover, it was probably that SAR of pH 3.5 and 2.5 input a large amount of H^+^ into the soil, resulting in consumption of the buffer system and the weakness of the regulation, so that the soil acidification intensified. When subjected to acid rain, a large amount of soil H^+^ could be absorbed by plant roots through root plasma membrane, and other soil H^+^ could react with anions (e.g., HCO_3_^−^, OH^−^, etc.) and organic acids (e.g., OA, MA, CA, etc.) secreted by plant roots. Wang et al.^41^ (2006) found that the rhizospheric pH of wheat treated with aluminum was positively correlated with root organic acids secretion. Figure 4D and Table 1 showed that treated with SAR at the same acidity level, the rhizospheric soil pH of Masson pine family No. 35 was significantly higher than that of family No. 79. In addition, the Pearson’s correlation coefficients between rhizospheric soil pH and chlorophyll *a*/*b* (− 0.860**), relative permeability of plasma membrane (− 0.943**), oxalic acid (− 0.924**) indicated that rhizosphere pH was significantly negatively correlated with these physiological properties (*P* < 0.01, Table 2). Treated with the SAR at the same pH, the root plasma membrane permeability of family No. 35 was lower than that of family No. 79, so that it could prevent more soil H^+^ from entering the roots and transport more anions to react with the soil H^+^. Furthermore, the organic acids secreted from family No. 35 was much more than that of family No. 79, so that it could bind to soil H^+^ better. These could explain why the rhizospheric pH of family No. 35 was higher than that of family No. 79 when subjected to SAR at the same pH. It could be proved by the results that the relative permeability of root plasma membrane and root oxalic acid secretion were instrumental in the resistance of Masson pine responding to acid stress.

The results in the correlation analysis (Table 2) of the test indicators indicated that the root plasma membrane permeability and oxalic acid secretion are negatively correlated with the morphology indexes, photosynthetic traits (except chlorophyll *a*/*b*), and soil pH of Masson pine families treated with SAR of different pH. Furthermore, the chlorophyll a/b, root plasma membrane permeability, and oxalic acid secretion were characterized as the principal components 1 (F1 = 76.33%, Fig. 5) by high negative scores according to PCA and were grouped as G-3 due to the negative correlations with the SAR pH based on the cluster analysis (Fig. 6). Therefore, the root plasma membrane permeability and root oxalic acid secretion of Masson pine can be considered as the key physiological factors to further study the pine root physiological mechanism resisting to acid stress.

## Conclusion

The effects of SAR on the growth and root physiological processes of the 16 pine families were studied in this paper. Herein, we focus on elucidating the relationship between acid resistance of Masson pine families and root physiological processes. The results evidently proved that Masson pine could tolerate the SAR at pH from 4.5 to 5.6, but there was significant difference in acid resistance among the pine families. Masson pine family No. 35 resisted to acid stress, but families Nos. 79, 114 and 116 showed acid sensitivity. Based on the data obtained, family No. 35 had better morphological characters, much more chlorophyll content and organic acids secretion, lower root plasma membrane permeability, and higher rhizospheric soil pH compared with family No. 79. Additionally, root plasma membrane permeability and organic acids secretion (especially oxalic acid) contribute greatly to the acid resistance of Masson pine. Understanding how plant regulators play a role in acid resistance mechanism of Masson pine and exploring their interaction is of great significance to the cultivation of Masson Pine under acid deposition. In future studies, the natural genetic variation of Masson pine in response to acid stress should be further studied, and its acid resistance mechanism, including root physiological process, metabolic system, enzyme system, as well as genetic regulation needs to be established.

## Methods

### Experimental soil

The woodland soil was collected at a soil depth of 0–20 cm from Nanjing Laoshan. The soil sample was sifted by 1 mm screen after being air dried, and base fertilizers containing of 0.2 g N (urea), 0.1 g P_2_O_5_ (KH_2_PO_4_) and 0.1 g K_2_O (K_2_SO_4_) were provided per kilogram of the soil, which were mixed and aged for 1 week and spared to be used. The soil physicochemical properties were shown in Supplementary as Table S3.

### Preparation of simulated acid rain

Based on the monitoring data of acid rain in Nanjing from April to July 2017 and the average concentrations of anions and cations in natural precipitation in Southern China, H_2_SO_4_, HNO_3_ with KF, NaCl, Ca(NO_3_)_2_, Mg(NO_3_)_2_, and (NH_4_)_2_SO_4_ were used to prepare the SAR by gradual dilution method. The as-prepared ion concentrations were (μmol L^−1^): K^+^, 8; Na^+^, 21; Ca^2+^, 33; Mg^2+^, 5; NH_4_^+^, 44; Cl^−^, 21; F^−^, 8. During 1992–2003, the concentration of SO_4_^2−^ had an obvious decrease in Nanjing rainwater, while NO_3_^−^ increased markedly^42^. The molar ratio of H_2_SO_4_ to HNO_3_ in SAR was set as 4:1. Referring to the maximum rainfall acidity (pH 2.1) that has emerged in China^43^ and the annual average pH value of acid rain in Jiangsu Province (pH 4.93) in 2017, the pH of SAR was set at 3 levels: pH 4.5 (mild acidification), pH 3.5 (moderate acidification), and pH 2.5 (severe acidification); pH 5.6 was used as the control condition. Three Masson pine per family were cultivated in each treatment to conduct three repetitions.

### Cultivation of Masson pine seedlings and sample collection

Sixteen families of Masson pine seeds collected from Jiangsu, Zhejiang, and Sichuan were used with the designated numbers of 35, 38, 76, 78, 79, 80, 88, 89, 90, 114, 115, 116, 117, 119, 147 and 151, respectively^44^. The seeds were sterilized with 75% ethanol for 10 min, and then washed with deionized water for 5 times with 20 min each. A 24 h soak time was allowed in 40 °C warm water, and subsequently the seeds were seeded on wet cotton and placed in incubator at constant 25 °C for germination. The 10-day healthy seedlings after germination were selected to cultivate with soil in the experimental rhizobox (Supplementary, Fig. S1). The moisture content of cultivation soil was kept at about 60% during the growth of Masson pine. The spray amount, time and frequency of SAR were implemented according to the precipitation status of Nanjing from April to July, 2017. The sowing time of Masson pine seeds was 20 April, 2018, and the times of germination and seedlings transfer were on April 29 and May 15, 2018, respectively. The next day after transplantation, the survival condition was checked and replanted immediately to ensure a survival rate of transplantation was ≥ 99.0%. These seedlings grew in a sunlight chamber. Considering the rapid growth rate of Masson pine in seedling stage and the obvious effect of environmental stress, the experiments were conducted on August 22, 2018, and the samples were collected after 100-day treatments.

Three seedlings of each Masson pine family per treatment were harvested and were carefully washed with tap water and deionized water three times, respectively. After that, the pine seedlings were separated into roots, stems, and needles. Rhizospheric and non-rhizospheric soils were excavated down to the same horizon (20 cm) in the rhizobox for separation analyses.

### Analytical methods

#### Morphology indexes

Plant samples were taken from each treatment, the root length and shoot length were measured, and the number of primary lateral roots with the length ≥ 2 cm grown from the main root was counted directly. Root samples were oven-dried at 60 °C until reaching a constant weight to measure the root dry weight.

#### Chlorophyll content

The contents of chlorophyll *a* and chlorophyll *b* were determined by spectrophotometry at 663 nm and 645 nm, respectively^45^. Samples from each family were selected randomly and the mid-upper needles on the sunny side of which were mixed. Exact 1 g sample was taken to be measured by UV-2401PC UV–visible spectrophotometer (Japan Shimadzu Company) after being extracted with 100 mL of acetone; repeated 3 times per family.

#### Relative permeability of roots plasma membrane

The relative permeability of roots plasma membrane was determined by conductivity method according to Inone and Kinoshita^46^ (2017). Fresh root of 0.1 g was washed with deionized water and cut into small pieces with length of 1 cm. Then, the root sample was put into a 20 mL stoppered test tube added with 10 mL of deionized water and oscillated in a shaking incubator for 30 min. The DDS-11C digital electric conductivity meter (Shenzhen Shengbang Electrical Appliance Factory, Tianjin, China) was used for determination of electric conductivity *E*_1_; then it was treated in boiling water for 10 min and the electric conductivity *E*_2_ was determined after cooling down; the background electric conductivity *E*_0_ was also measured. The relative plasma membrane permeability was calculated as: *p* (%) = (*E*_1_ − *E*_0_)/(*E*_2_ − *E*_0_) × 100%. The degree of membrane damage was calculated according to the following formula: the degree of membrane damage (*p*/%) = (*P*_*T*_ − *P*_*CK*_) × 100%, where *P*_*T*_ was the relative plasma membrane permeability of the treatments; *P*_*CK*_ was the relative plasma membrane permeability of the control.

#### Determination of organic acids

The ultra-trace organic acids were determined by an accelerated solvent extraction-solid-phase extraction-liquid chromatography with electrospray ionization-tandem mass spectrometry methodology according to Wang et al.^47^ (2015). The detailed data were shown in Supplementary as Table S1.

#### Soil pH

After 100 days of seedling cultivation, the rhizospheric and non-rhizospheric soil samples (Supplementary, Fig. S1) were extracted by 1 mol L^−1^ KCl (2.5:1 water/soil ratio). The soil pH was measured by a PHS-25 digital acidity meter (Shanghai Precision Instrument Co., Ltd.) according to Yao et al.^48^ (2019). The detailed data were shown in Supplementary as Table S2.

### Statistical analysis

Data were processed by One-Way ANOVA, Pearson’s correlation coefficients, principle component analysis, and cluster analysis using SPSS 11.5 software. The factor in one-way ANOVA was the Masson pine family and the significance (*P* < 0.05) of differences among the Masson pine families was evaluated by Duncan’s multiple range tests; tabulation and plotting were performed by Excel software and Origin Pro Version 2017 (OriginLab Corporation, Northampton, MA, USA).

## Supplementary Information

Supplementary Information.
