# Supplementary material for: Acid resistance of Masson pine (Pinus massoniana Lamb.) families and their root morphology and physiological response to simulated acid deposition
Source: Sci Rep. 2020 Dec 16;10:22066. doi: 10.1038/s41598-020-79043-1 (PMC7744545; doi:10.1038/s41598-020-79043-1)
Supplement: Supplementary file 1 — Supplementary Information. [file 41598_2020_79043_MOESM1_ESM.docx]

**Acid resistance of Masson pine (*Pinus massoniana* Lamb.) families and their root morphology and physiological response to simulated acid deposition**

Sijie Zhou^1,2^, Min Zhang^1,2^, Shuzhan Chen^1,2^, Wen Xu^1,2^, Liting Zhu^1,2^, Shurui Gong^1,2^, Xiaoqin He^1,2^ & Ping Wang^1,2^

^1^ College of Biology and the Environment, Nanjing Forestry University, Nanjing 210037, China.

^2^ Key Laboratory of Forest Genetics & Biotechnology of Ministry of Education, Nanjing Forestry University, Nanjing 210037, China.

Sijie Zhou: 380191549@qq.com

Min Zhang: 694663104@qq.com

Shuzhan Chen: 462396584@qq.com

Wen Xu: 870087587@qq.com

Liting Zhu: 1016340042@qq.com

Shurui Gong: 695411431@qq.com

Xiaoqin He: 1187061939@qq.com

Ping Wang: [18061713169@163.com](mailto:18061713169@163.com) (Corresponding author)

**Appendix A. Supplementary data**

**Table S1.** The organic acids secreted from the Masson pine roots exposed to simulated acid rain (SAR) at different pH

| SAR pH | Organic Acid (*w*/ng L^-1^) | | | | | | | | | |
| --- | --- | --- | --- | --- | --- | --- | --- | --- | --- | --- |
|  | (a) No.35 Masson pine family | | | | | (b) No.79 Masson pine family | | | | |
|  | Oxalic Acid | Malic Acid | Citric Acid | Succinic Acid | Acetic Acid | Oxalic Acid | Malic Acid | Citric Acid | Succinic Acid | Acetic Acid |
| 2.5 | 1251.93 ± 67.55 | 183.11 ± 9.42 | 83.46 ± 10.32 | ND | ND | 1087.36 ± 82.51 | 112.73 ± 7.77 | 69.54 ± 7.32 | ND | ND |
| 3.5 | 1073.27 ± 58.73 | 272.70 ± 13.43 | 104.38 ± 11.11 | ND | ND | 934.86 ± 55.41 | 214.63 ± 8.57 | 83.71 ± 6.77 | ND | ND |
| 4.5 | 482.79 ± 32.72 | 207.44 ± 11.46 | 87.40 ± 10.34 | ND | ND | 375.98 ± 32.65 | 159.22 ± 7.44 | 71.24 ± 6.57 | ND | ND |
| 5.6 | 166.54 ± 25.58 | 112.83 ± 10.36 | 71.56 ± 6.78 | ND | ND | 138.14 ± 22.42 | 95.57 ± 6.83 | 60.73 ± 4.86 | ND | ND |

Note: Results are expressed as mean ± standard error (n = 3). ND = not detected.

**Table S2.** The rhizosphere and non-rhizosphere pH for Masson pine families No.35 and No.79 exposed to SAR at different pH

| SAR pH | Soil pH | | | |
| --- | --- | --- | --- | --- |
|  | (a) Masson pine family No.35 | | (b) Masson pine family No.79 | |
|  | Rhizosphere | Non-Rhizosphere | Rhizosphere | Non-Rhizosphere |
| 2.5 | 3.79 ± 0.23 | 3.35 ± 0.31 | 3.18 ± 0.26 | 2.96 ± 0.27 |
| 3.5 | 4.52 ± 0.32 | 4.17 ± 0.24 | 4.02 ± 0.32 | 3.77 ± 0.28 |
| 4.5 | 5.23 ± 0.41 | 5.08 ± 0.21 | 5.12 ± 0.24 | 4.94 ± 0.37 |
| 5.6 | 5.96 ± 0.21 | 5.92 ± 0.33 | 5.93 ± 0.32 | 5.86 ± 0.22 |

Note: Results are expressed as mean ± standard error (n = 3).

**Table S3.** The soil physicochemical properties

| Physicochemical properties | |
| --- | --- |
| Cation exchange capacity (CEC, *w*/mol kg^-1^) | 6.28 |
| Organic carbon (OC, *w*/g kg^-1^) | 22.3 |
| water holding capacity (%) | 36 |
| Clay (%) | 15.3 |
| Soil pH (H_2_O) (the ratio of water: soil = 2.5:1) | 6.65 |
| Total nitrogen (TN, *w*/g kg^-1^) | 2.12 |
| Total phosphorus (TP, *w*/g kg^-1^) | 0.893 |
| Total potassium (TK, *w*/g kg^-1^) | 13.6 |
| Total aluminum (*w*/g kg^-1^) | 65.7 |
| Exchangeable Al (*w*/g kg^-1^) | 0.72 |
| Exchangeable Fe (*w*/g kg^-1^) | 34.2 |
| Exchangeable Ca (*w*/g kg^-1^) | 1.41 |


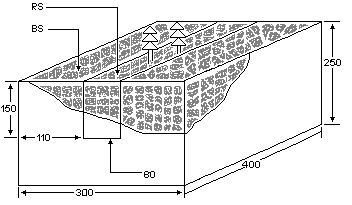


**Figure S1.** Schematic diagram of the experimental rhizobox, in which the 80-mm thick soil layer between the two sheets of nylon mesh was considered as the rhizosphere soil (RS). Soil far beyond the central compartment represents the bulk soil (BS). That is, non-rhizosphere soil.

**Figure S2.** The morphology indexes, (A) root length (*l*/cm), (B) shoot length (*l*/cm), (C) root dry weight (*m*/mg), and (D) primary lateral root number of Masson pine families exposed to the SAR at pH of 2.5, 3.5, 4.5 and 5.6. Based on Duncan’s multiple range tests with *P* < 0.05, significant differences between Masson pine families are represented by lowercase letters. Results are expressed as mean ± standard error (n = 3).

**Figure S3.** Contents of (A) chlorophyll a (*w*/mg g^-1^), (B) chlorophyll b (*w*/mg g^-1^), (C) chlorophyll a+b (*w*/mg g^-1^), and (D) the value of chlorophyll a/b for the Masson pine families exposed to the SAR at pH of 2.5, 3.5, 4.5, and 5.6, respectively. Based on Duncan’s multiple range tests with *P* < 0.05, significant differences between Masson pine families are represented by lowercase letters. Results are expressed as mean ± standard error (n = 3).

**Figure S4.** (A) The relative permeability (*p*/%) and (B) damage degree (*p*/%) of root plasma membrane of sixteen Masson pine families exposed to the SAR. The value of membrane damage degree of Masson pine families at pH 5.6 is 0, which is not shown in Fig. S4B. Based on Duncan’s multiple range tests with *P* < 0.05, significant differences between Masson pine families are represented by lowercase letters. Results are expressed as mean ± standard error (n = 3).
